# Positive Affect Moderates the Relationship Between Salivary Testosterone and a Health Behavior Composite in University Females

**DOI:** 10.1007/s12529-019-09824-0

**Published:** 2019-12-04

**Authors:** Luci A. Martin, Mariam Ter-Petrosyan

**Affiliations:** Psychology Department, University of La Verne, 1950 3rd Street, La Verne, CA 91750 USA

**Keywords:** Positive affect, Negative affect, Salivary testosterone, Health behavior

## Abstract

**Background:**

Testosterone is released in both men and women and plays an important role in social functioning and motivation. Greater testosterone in women has been associated with negative physical health outcomes, while lower testosterone has been associated with psychological disorders. The following cross-sectional study examined the contribution of salivary testosterone, positive and negative affect, and demographic variables in predicting a composite health behavior score (cigarette use, hours of sleep, fruit/vegetable intake, following an exercise routine).

**Method:**

The sample (mean age 21.17, *SD* = 6.13) consisted of 87 female university students asked to complete a demographic and lifestyle behavior questionnaire, the Positive and Negative Affect Schedule, and provide a saliva sample. Participants self-identified as Latina (37.9%), European American (32.2%), Asian American (5.7%), African American (4.6%), or Mixed/other (19.5%). Hierarchical regression analyses were used to examine whether positive and negative affect served as a moderator between salivary testosterone and a health behavior composite.

**Results:**

Results indicated that positive affect moderated the relationship between salivary testosterone and the composite health behavior score (*t* = − 2.42, *p* = .018, Adj. *R*^2^ = .21, *F* (5, 81) = 5.07, *p* < .001) such that the healthiest behaviors were observed in participants with high positive affect and low salivary testosterone. Findings remained after adjusting for oral contraceptive use, income level, relationship status, and ethnicity.

**Conclusions:**

These results provide a preliminary foundation for future research examining the interplay of neuroendocrine function, psychological factors (i.e., positive affect), and behavior. Further empirical studies can focus on expanding this research in larger, representative samples.

## Introduction

Testosterone is a hormone released as the end product of the hypothalamic-pituitary-gonadal axis [1]. Testosterone operates under a diurnal cycle such that greater quantities are released upon awakening and decline throughout the day [2]. In men, testosterone is produced in the testes, while in women, it is produced by the adrenal cortex and ovaries [1]. Testosterone plays a key role in the metabolism of carbohydrates, proteins, and fat [3]. Altered levels of testosterone have been consistently reported in patients with a number of life-threatening diseases. For example, reduced levels of testosterone are found in men with obesity and/or type 2 diabetes [3], while elevated levels of testosterone are associated with an increased risk of breast cancer in women [4].

Salivary testosterone has become a popular biomarker in psychophysiological research given its ease of assessment and relationship to both adaptive (i.e., exercise, sexual functioning, and prosocial interactions) and maladaptive (i.e., substance use, risky sexual, and aggressive) behaviors [1, 5]. Studies typically involve experimental designs that examine causal effects of testosterone administration (i.e., [6]) or correlational designs that examine psychological characteristics (i.e., personality) with basal testosterone as a trait variable (i.e., [7]). Though typically thought of as a male sex hormone psychologically related to aggression and risky or impulsive behavior, testosterone is released in men and women and appears to play an important role in social functioning and prosocial motivation for both sexes [8]. Historically, salivary testosterone has been excluded from studies in women due to concern that the quantity of testosterone in saliva was too low to detect and menstrual cycle fluctuations would make collection of accurate samples difficult [9]. Across age groups, the mean salivary testosterone levels of men are approximately six times greater than those of women [10]. Salivary testosterone decreases 1.0–1.5% annually in both men and women across age groups and the level of testosterone in saliva is reported to fall below detection levels at the 2.5th percentile in women age 52 and above [10]. Moderate to large effect sizes have been noted between salivary testosterone and serum testosterone levels in women (i.e., [11–13]). Salivary testosterone appears to provide a valid estimate of testosterone levels in women, particularly in non-clinical samples below age 52.

Growing interest in the role of testosterone in health has led to an increase in studies in women and to the discovery that testosterone functions quite differently physiologically in women than in men. Testosterone is positively associated with weight and fat deposition in women, though inversely related in men [5]. In post-menopausal women, a significant decrease in body mass index was associated with a significant decrease in testosterone levels across a 5- to 7-year timespan [14]. Exercise has been associated with both increases and decreases in testosterone in females, depending on intensity, specific activity, and duration (i.e., [5, 15]). Again, women with higher testosterone levels have been shown to be at an increased risk of breast cancer [4]. These findings suggest that increased testosterone levels in women can be summarized as placing women at risk for disease, while the inverse may be true for men [3]. Despite potential for adverse health outcomes associated with greater levels of testosterone, testosterone has a number of beneficial social and psychological effects.

The Positive Affective Neuroendocrinology perspective proposed by Welker, Gruber, and Mehta [1] provides a synthesis of research on the connection between testosterone, behavioral dysregulation via reward motivation, and affect. Specifically, the theory argues that testosterone increases reward processing, motivation, and positive affect, which leads to behavioral risk-taking behaviors. As an example, patients with bipolar disorder have been shown to demonstrate higher levels of testosterone during manic episodes [16, 17], which are characterized by impulsive, risky behaviors [18]. Testosterone has largely been tied to approach-avoidance behavioral paradigms, with greater testosterone levels being associated with approach (reward) behaviors, while lower levels of testosterone are associated with avoidance (punishment) [1, 19]. Approach motivation is typically associated with positive affect, while avoidance behaviors are associated with negative affect [20].

As with physiological differences, many studies have revealed that testosterone does not operate in the same way “psychologically” for women as it does for men. Ultimately, testosterone assists in allowing individuals to seek and sustain social status [8], which often differs among men and women. As an example, the relationship between sexual desire and testosterone differs based on the sex/gender of the participants and other contextual factors [21]. Testosterone has been found to be inversely related to experimentally manipulated aggression in healthy female participants and negatively associated with basolateral amygdala reactivity in the presence of angry faces, suggesting a fear-reducing, prosocial function [22]. Exogenous testosterone induced in females has been associated with an increase in positive affect [6]. Finally, in a large, longitudinal cohort study, testosterone levels were lower in females who met criteria for depressive disorder, generalized anxiety disorder, social phobia, and agoraphobia [23]. In summary, greater testosterone appears to be associated with healthier psychological outcomes in women.

The purpose of the current cross-sectional study was to examine the relationships among salivary testosterone, positive and negative affect, and a composite health behavior measure in a sample of female university students. In order to explore these relationships, the following hypotheses were generated: (1) given the associations cited between disease and greater testosterone levels (i.e., [4, 14]), it is expected that lower testosterone levels will be associated with health-promoting behaviors in female university students; (2) given the associations cited between affect or mood disorders (i.e., [6, 23]), it is expected that testosterone levels will be positively associated with positive affect, while inversely associated with negative affect; and (3) it is expected that positive and negative affect will moderate the relationship between testosterone and health behavior, though the direction is not predicted.

### Participants

Participant eligibility included: (1) enrollment in an undergraduate course; (2) 18 years of age or older; and (3) fluency in written and spoken English. No exclusion criteria were implemented in the recruitment process. In exchange for completion of the study, participants were awarded class credit by a designated instructor for their participation, a $10 gift card or a relaxation and stress reduction workbook.

A sample of 107 university students (83% female) were recruited to participate in a study. Given the small sample of male participants (*n* = 18), limited power to detect differences, and significant differences between salivary testosterone levels in males (*M* = 118.70, *SD* = 31.59) and females (*M* = 44.68, *SD* = 23.54) within the sample (*t*(101) = 10.93, *p* < .001), males were excluded from further analyses. This resulted in a sample of 89 female participants; however, one participant did not provide a saliva sample and one participant did not provide a sufficient amount of saliva for analysis. This resulted in a final sample of 87 female participants.

The average age of the participants was 21.17 (*SD* = 6.13, range = 18–63). Two participants were above the age of 32 years (i.e., 49 years and 63 years); however, no differences were detected by eliminating these two participants; therefore, they were retained. Participants self-identified as European American (32.2%), Latina American (37.9%), African American (4.6%), Asian American (5.7%), biracial or mixed (14.9), and other (4.6%). Approximately 84% identified as heterosexual. Most (66.7%) of the sample reported an annual income of less than $10,000. As an indicator of socioeconomic status, 43.7% of participants reported that their mother had received less than or equal to a high school equivalency, while 46% reported that their father had received less than or equal to a high school equivalency. Fifty-two percent of participants reported that they were in a relationship.

### Procedure

The study was approved by and adhered to institutional review board requirements on the use of human subjects. The participants logged into a computerized system (the SONA system), signed up for the study, and scheduled their appointment time. Upon scheduling an appointment with the principal investigator, an email was sent to the participant immediately and 24 h prior to the testing session reminding them of the session and to provide guidelines for a reliable physiological sample. In order to avoid blood and other oral contaminants, participants were provided with instructions to avoid a major meal within 60 min, avoid alcohol consumption for 24 h, avoid eating dairy products, avoid acidic or high sugar foods, to not brush teeth within 3 h, to wash mouth out with water 10 min prior, and to document all prescription and over-the-counter medications taken prior to sample collection. Upon arrival, participants were again reminded of these instructions verbally and also provided a cup of water prior to participation.

Sleep and other contextual factors have been shown to influence the diurnal fluctuation of testosterone (i.e., [2, 24]), making afternoon sampling an ideal time of assessment [5]. Participants were scheduled between 1400 and 1600 h or 1200 and 1400 h, to accommodate student participant and facilitator schedules. The participants met in designated research rooms on the university campus in small groups of one to five. Participants were asked to provide written informed consent, complete a questionnaire, and then provide a saliva sample. To examine the relationship among testosterone as a trait characteristic with psychological variables, assessment at one time point has been recommended, given its stability across days and weeks (i.e., [2, 5]). Following participation, participants were thanked and handed a debriefing form with contact information for health and wellness resources on campus and in the community.

### Measures

#### Background Questionnaire

Participants were asked to self-report gender, ethnicity, age, income level, relationship status of self and parents, sexual orientation, education level of self and parents, living arrangement, number of children, social organization involvement, and whether spiritual or religious.

#### Lifestyle Behavior and Health History Questionnaire

Participants were asked several health-related questions known to be associated with disease outcomes. Question stems and response options were adapted from the Behavioral Risk Factor Surveillance System Questionnaire [25] and research documenting the association of each behavior with health outcomes (i.e., [26]). Lifestyle behaviors were assessed using dichotomous, categorical and open-ended response formats. Specifically, participants were asked: “Do you follow a regular exercise routine?” with the option “yes” or “no;” How many hours of sleep do you typically get each night?” with the option to endorse “less than 5, 5–6, 7–8, 9 or more;” “How many cigarettes do you smoke each day?” with the response options “0, 1–10, 11–20, 20 or more” and to check whether the following statement best describes typical eating habits: “Eat 3–5 fruits and/or vegetables a day.” A composite health behavior score was created to account for the tendency of lifestyle behaviors to cluster together (i.e., [27, 28]) and to reduce the likelihood of type 1 error [29]. Lifestyle behaviors with strong evidence linking each to mortality (i.e., cigarette use [26], fruit/vegetable intake [30], sleep [31], and exercise [26]) were dichotomized such that each question was given a point if the person engaged in the behavior in a healthy manner. Therefore, participants who did not use cigarettes, who checked eating 3–5 fruits and/or vegetables a day, reported typically sleeping 7–8 h a night, and/or endorsed engaging in a regular exercise routine were given a point for each of these behaviors. This resulted in a composite health behavior score ranging from 0 to 4.

#### Positive and Negative Affect

The Positive and Negative Affect Schedule [32] was used to assess positive and negative affect. Ten items were used to calculate positive affect and 10 items were used to calculate negative affect. Items were assessed on a 5-point Likert-type scale ranging from 1 (*very slightly or not at all*) to 5 (*extremely*). Participants were asked to respond according to how they had felt over the past few weeks. According to the manual [32], internal consistency values for the positive and negative affect scales ranged from a reported 0.86 to 0.87, with a low intercorrelation (*r* = 0.09). Test-retest reliability across a 2-month retest interval was *r* = 0.43 for positive affect and *r* = 0.41 for negative affect and remained high (i.e., *r* = 0.42 and 0.43) at a mean interval of 72.4-month follow-up. Construct validity for the positive and negative affect scales was demonstrated through correlations with the NEO-Five Factor Inventory, General Temperament Survey, Eysenck Personality Questionnaire, and other state and trait measures [32]. Internal consistency within this sample was α = .87 for positive affect and α = .84 for negative affect.

#### Salivary Basal Testosterone

The SalivaBio Oral Swab (SOS) Saliva Collection Method was used to collect saliva samples to calculate salivary testosterone levels. The samples were placed in a Salimetrics cryostorage box and then stored in a freezer (the temperature remained below – 20 °C throughout the study). All collected samples were transferred to Salimetrics (https://www.salimetrics.com/) for analysis and properly disposed of following completion of the study. Samples were tested for salivary testosterone using a high-sensitivity enzyme immunoassay. On the day of the assay, samples were thawed to room temperature, vortexed, and centrifuged for 15 min at approximately 3000 rpm (1500×*g*). The test used 25 μL of saliva per determination and had a lower limit of sensitivity of 1 pg/mL, an average intra-assay coefficient of variation of 4.6%, and an average inter-assay coefficient of variation of 9.85%, as per the kit manufacturer. The Salimetrics SalivaLab acceptance criteria for duplicate hormone results require a coefficient of variation < 15% between samples 1 and 2.

### Data Analysis

All data were analyzed using IBM SPSS Statistics 25 [33]. All self-reported survey data were entered by a graduate research assistant and checked by a second graduate research assistant and the primary investigator. Errors were corrected by comparing the data file to the original surveys. Frequency and descriptive statistics were calculated. Preliminary analyses were conducted to examine that all variables were within an acceptable range. Skewness and kurtosis values were examined to determine if continuous variables were normally distributed, using a cut-off of ± 1. Data that were not normal were transformed. A correlation matrix was conducted to examine bivariate relationships and to determine multicollinearity of predictor variables using a cut-off of *r* = .70. Additional analyses (e.g., independent samples *t* tests, chi-square test for independence, analysis of variance) were conducted to both understand the data and determine if covariates were present. A hierarchical multiple regression model was used to examine whether salivary testosterone and positive and negative affect (independent variables) predicted the health behavior composite (dependent variable). Using methods outlined by Aiken and West [34], continuous independent variables were centered and interaction terms were multiplied by one another, prior to entering in the model. Tolerance, variance inflation factors, the normal probability plot of the regression standardized residual and the scatterplot of the standardized residuals were examined to assess for violations of assumptions. Significant interaction terms were plotted at values of one standard deviation above and below the mean for each independent variable. A *p* value of .05 was used to determine significance.

## Results

### Preliminary Analyses

A total of 87 female participants completed all self-report questionnaires and provided a saliva sample sufficient for analyses (see Tables 1 and 2). A between-subject analysis of variance was conducted to examine testosterone across time and academic year (1400–1600 h (*n* = 58) or 1200–1400 h (*n* = 29). There was no statistical difference based on time for testosterone *F*(1, 85) = 2.38, *p* = .13). Seasonality or time of year has also been noted to demonstrate effects on testosterone levels in prior research (i.e., [5]); therefore, season was examined. No statistical differences were noted by season (fall (*n* = 37) or spring (*n* = 50), *t*(85) = − 0.22, *p* = .83). Therefore, participants were combined across academic years and semesters. Testosterone was naturally log-transformed to account for the lack of a normal distribution.

**Table 1:** Means, standard deviations, and ranges of continuous variables (*n* = 87)

Variable	Mean	Standard deviation	Range	Skewness	Kurtosis
Age	21.17	6.13	18-63	5.04	29.56
Body mass index	24.94	5.52	14.06–43.43	.83	.78
Positive affect	3.04	.74	1.00–4.70	− .56	.29
Negative affect	2.10	.72	1.10–4.20	.58	− .18
Testosterone (pg/mL)	44.68	23.54	6.93–164.79	1.81	6.80
LnTestosterone	3.67	.53	1.94–5.10	− .47	.92
Health behavior composite	2.49	.87	1.00–4.00	.13	− .64

Health behavior composite includes cigarette use, exercise routine, fruit/vegetable intake, and hours of sleep. *lnTestosterone*, natural log transformation of salivary testosterone

**Table 2:** Frequency statistics for dichotomous and categorical variables (*n* = 87)

Variable	Response options	%
Ethnicity	Hispanic/Latina	37.9
	White/European American	32.2
	African American/Black	4.6
	Asian/Asian American	5.7
	Mixed	14.9
	Other	4.6
Financial income per year	Less than $10,000	66.7
	$10–19,000	16.1
	$20–39,000	10.3
	$40–59,000	5.7
	$80,000 or above	1.1
Employment status	Part time (less than 30 h per week)	50.6
	Not employed	43.7
	Employed full time	5.7
Relationship status	In a relationship	51.7
	Not in a relationship	48.3
Sexual orientation	Heterosexual	83.7
	Lesbian	5.8
	Bisexual	7.0
	Questioning	3.5
Religious	Yes	47.1
	No	52.9
Cigarette use	Yes	2.3
	No	97.7
Exercise routine	Yes	62.1
	No	37.9
Hours of sleep per night	Less than 5	5.7
	5–6	47.1
	7–8	46.0
	9 or more	1.1
Eat 3–5 fruits and/or vegetables per day	Yes	43.7
	No	56.3
Health behavior composite	1	11.5
	2	41.4
	3	33.3
	4	13.8

Health behavior composite includes cigarette use, exercise routine, fruit/vegetable intake, and hours of sleep

### Health Status of Participants

For health-related behaviors and history, 43.7% of the sample endorsed eating 3–5 fruits/vegetables per day, 62.1% endorsed having a regular exercise program, and 46% endorsed getting an average of 7–8 h of sleep per night. Two participants endorsed smoking one cigarette per day. Participants endorsed an average of 2.49 health behaviors (*SD* = 0.87, range 1–4). Twenty-six percent of the sample checked that they visited a physician less than once per year. For history of medical conditions for self or family, 63.2% endorsed hypertension, 25.3% heart attack/myocardial infarction, 23% stroke, 69% diabetes, 57.5% high cholesterol, 37.9% obesity, 43.7% anxiety, 18.4% bipolar disorder, and 43.7% depression. The sample had a self-reported mean body mass index of 24.94 (*SD* = 5.52, range = 14.06–43.43); 53.4% of the sample were within a normal range, while 6.8% were classified as underweight and 39.8% as overweight or obese. Thirty-five percent of participants reported prescription medication use, with 20.7% reporting use of oral contraceptives.

### Covariates

Participants currently in a relationship reported lower testosterone (*t* = 2.45, *p* = .02). Participants with an income level of less than $10,000 reported lower testosterone than those who reported an income of $10,000 or greater (*t* = − 2.2, *p* = .03). Testosterone (*t* = 3.72, *p* < .001) was lower in participants who endorsed use of oral contraceptives than in those who did not. Oral contraceptive use was also differentiated by ethnic category (*χ*^2^ = 13.74, *p* < .001), such that zero Latina participants endorsed use of oral contraceptives. Latina participants endorsed significantly less hours of sleep on a typical night than other ethnic groups (*t* = 2.59, *p* = .01). Age, sexual orientation, and body mass index were not associated with positive affect, negative affect, or salivary testosterone. Given these differences, analyses were adjusted for Latina ethnicity, oral contraceptive use, relationship status, and income level.

### Bivariate Relationships Among Salivary Testosterone, Affect, and Behavior

Pearson product moment correlations were conducted on continuous variables of interest (see Table 3). All significant relationships remained after adjusting for oral contraceptive use, ethnicity, income, and relationship status.

**Table 3:** Unadjusted and adjusted correlations among positive affect, negative affect, salivary testosterone, and a composite health behavior score (*n* = 87)

Variable	1	2	3	4
1. Positive affect	-			
2. Negative affect	− .27**	-		
3. Testosterone	− .14	− .05	-	
4. Health behaviors	.36**	− .23*	.08	-
Adjusted for covariates (Latina, relationship status, income, oral contraceptive use)				
1. Positive affect	-			
2. Negative affect	− .28*	-		
3. Testosterone	− .12	− .05	-	
4. Health behaviors	.36**	− .25*	.11	-

*Testosterone*, natural log transformation of salivary testosterone; *health behaviors*, health behavior composite score (cigarette use, exercise routine, fruit/vegetable intake, hours of sleep)

*p < .05; **p < .01

Given the distribution of health behaviors across the health behavior composite score, analysis of variance was used to see whether changes in salivary testosterone or affect changed with the addition of each behavior. The analysis of variance model using testosterone as the dependent variable was not significant. A multivariate analysis of variance using positive affect and negative affect as dependent variables was significant (*F*(6, 164) = 3.36, *p* = .004, partial eta squared = .11). Inspection of each dependent variable revealed that the relationship was significant for positive affect (*F*(3, 83) = 5.51, *p* = .002), but not for negative affect (*F*(3, 83) = 2.55, *p* = .06). Using Tukey’s HSD post hoc analyses, significant differences were present between those who reported one health behavior (*M* = 2.36, *SD* = .87, *n* = 10) and three (*M* = 3.31, *SD* = .46, *n* = 29, *p* = .002) as well as between one health behavior and four (*M* = 3.28, *SD* = .70, *n* = 12, *p* = .014).

### Multivariate Relationships Among Salivary Testosterone, Affect, and Behavior

**Fig. 1:**
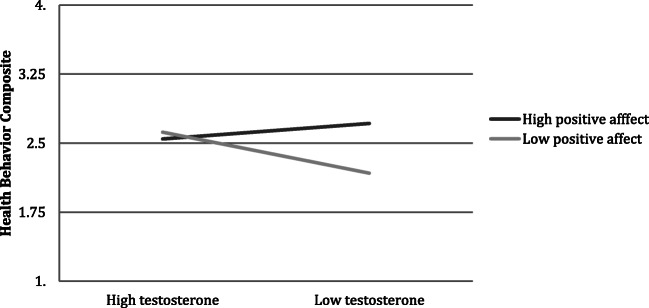
Positive affect moderates the relationship between salivary testosterone and a health behavior composite (cigarette use, exercise routine, fruit/vegetable intake, hours of sleep) (*n* = 87)

A hierarchical multiple regression model was used to explore the relationship between salivary testosterone, positive and negative affect, and the composite health behavior score (Table 4). Positive affect moderated the relationship between testosterone and the composite health behavior score (*t* = − 2.42, *p* = .018, Adj. *R*^2^ = .19, *F* (5, 81) = 5.07, *p* < .001) such that the highest number of health behaviors was among individuals low in testosterone, but high in positive affect (2.77 health behaviors) and lowest for those low in both positive affect and testosterone (2.30 health behaviors). Findings remained after adjusting for oral contraceptive use, income level, relationship status, and ethnicity (see Fig. 1).

**Table 4:** Regression model for salivary testosterone, positive and negative affect, and a composite health behavior score (*n* = 87)

Variable	Health behaviors				
	*B*	*SE B*	β	*t*	*p*
Block 1 (constant)	2.49	.09		28.60	.000
Negative affect	−.16	.13	− .13	− 1.24	.217
Positive affect	.41	.13	.34	3.26	.002
Testosterone	.21	.17	.12	1.22	.225
	Adj. *R*^2^ = .14, *F*(3, 83) = 5.48, *p =* .002				
Block 2 (constant)	2.45	.09		28.45	.000
Negative affect	− .16	.12	− .13	− 1.32	.192
Positive affect	.45	.12	.38	3.66	.000
Testosterone	.30	.17	.18	1.77	.081
Testosterone × PA	− .76	.32	− .24	− 2.43	.018
Testosterone × NA	− .34	.24	− .14	− 1.41	.161
	Adj. R^2^ = .19, *F*(5, 81) = 5.07, *p* < .001				
Block 3 (constant)	2.36	.17		13.66	.000
Negative affect	− .19	.13	− .16	− 1.52	.133
Positive affect	.42	.12	.36	3.48	.001
Testosterone	.36	.19	.22	1.88	.065
Testosterone × PA	− .77	.32	− .24	− 2.42	.018
Testosterone × NA	− .39	.25	− .16	− 1.56	.123
Oral contraceptives	.29	.25	.14	1.16	.252
Income	.25	.19	.14	1.31	.195
Relationship status	.02	.18	.01	.12	.909
Latina	− .18	.20	− .10	− .91	.364
	Adj. *R*^2^ = .21, *F*(9, 77) = 3.57, *p* = .001				

Health behavior composite includes cigarette use, exercise routine, fruit/vegetable intake, and hours of sleep

*Testosterone*, natural log transformation of testosterone; *NA*, negative affect; *PA*, positive affect

### Post Hoc Analyses

Three logistic regression analyses were conducted to explore whether the independent variables (salivary testosterone, positive affect, and negative affect) predicted three dichotomous individual health behaviors (exercise routine, fruit/vegetable intake, and hours of sleep). Cigarette use was not examined as only two participants endorsed cigarette use. For exercise routine, the model was significant (*χ*^2^ (3, *n* = 87) = 17.14, *p* = .001). The model explained between 17.9% (Cox and Snell *R* square) and 24.3% (Nagelkerke *R* squared) of the variance in exercise routine and correctly classified 73.6% of the sample. Positive affect (*B* = 1.36, Wald = 11.92, *p* = .001, odds ratio = 3.88) and salivary testosterone (*B* = 0.97, Wald = 4.12, *p* = .042, odds ratio = 2.64) were significant predictors of exercise routine, though the interaction among salivary testosterone and positive affect was not significant. The models for fruit/vegetable intake and hours of sleep were not significant at the *p* < .05 level.

## Discussion

The results of this study suggest that salivary testosterone and positive affect play an important role in the health of female university students. Most of the research examining testosterone’s role in health outcomes has focused on its relationship to risky behaviors and failed to consider the role of affect or health-promoting behaviors. Though positive affect and testosterone were not directly related in this sample, the interaction between positive affect and testosterone predicted a composite health behavior score. Ford, Zhao, Tsai, and Li [26] identified three health behaviors (never smoked, healthy diet, moderate physical activity) that demonstrate a dose-dependent relationship with all-cause mortality. The risk for all-cause mortality was reduced by 56% for non-smokers, 47% for the physically active, and 26% for those who reported a healthy diet [26]. Compared with individuals who engaged in zero behaviors, the risk for mortality was 82% lower in those who engaged in all three health or low-risk behaviors [26]. Targeting behavior change in young adults is important because behavioral changes at a young age are more likely to impact health than behavioral changes later in life (e.g., [35]). In the USA, among 12–19-year-olds, 36.9% are classified as overweight or obese, which increases to 63.7% among 20–39-year-olds [36]. Ideal levels of systolic blood pressure are present for about 88% of 12–19-year-olds, while only 68% of 20–39-year-olds [36]. Early identification of risk factors and intervention is necessary to prevent health conditions.

Hypothesis 1, which suggested that lower testosterone levels would be associated with health behaviors, was not supported. The relationships between testosterone and each of the individual health behaviors and the composite health behavior score were not significant. It is difficult to speculate why these relationships were not significant. It is likely that the adverse health outcomes associated with increased testosterone that have been noted in women (i.e., breast cancer; [4]) have not yet had the opportunity to have an impact on university-aged females. On the other hand, almost 40% of the sample were classified as overweight or obese, a characteristic that has been associated with increased testosterone levels [5]. Despite being overweight, the sample was quite healthy, with participants reporting an average of two of the four health behaviors. Hormones and behavior have a modulating effect. Engaging in health behaviors likely keeps hormone levels at an appropriate level, via a feedback loop utilizing the hypothalamic-gonadal axis, given that the organism’s goal is always to maintain homeostasis [37].

Hypothesis 2 was not supported, as testosterone levels were not significantly associated with positive or negative affect. This is inconsistent with several studies that have shown a significant relationship between testosterone and affect or mood. For example, exogenous testosterone induced in a small sample (*n* = 24) of university females increased positive affect, using the same measure as the current study, though the effect was only present 4 h after administration [6]. Large cohort studies have shown testosterone levels to be negatively associated with disorders suggestive of negative affect (i.e., depressive disorder, generalized anxiety disorder, social phobia, and agoraphobia; [23]), while positively associated with manic episodes [1]. Most of the research in this area has focused on samples with clinical diagnoses (i.e., mood disorders), which was not the focus of the current study. Mood and affect are different constructs that have differential effects on physiology. The positive affect scale included the following items: active, alert, attentive, determined, enthusiastic, excited, inspired, interested, proud, and strong [32]. None of these items warrants diagnosis of a clinical condition and, instead, represents emotions that suggest healthy psychological functioning.

Analyses to examine hypothesis 3 were significant. Positive affect moderated the relationship between salivary testosterone and the composite health behavior score such that greater positive affect and lower testosterone were associated with the greatest number of health behaviors, yet those low in both positive affect and testosterone reported the lowest number of health behaviors. In connection with the Positive Affective Neuroendocrinology perspective [1], one inference is that if an individual’s system is functioning properly, testosterone will increase positive affect, leading to an increase in motivation to pursue prosocial behaviors. The health behaviors examined (exercise, eating healthy, not smoking, and adequate sleep) all serve to increase success of the individual by increasing longevity [26], cognitive functioning and thus academic and professional success, and possibly attractiveness and mate selection. When the system does not work properly (i.e., testosterone does not increase positive affect), the individual is not motivated to pursue prosocial behaviors such as exercise, diet, and sleep, setting off a series of long-term psychological, social, and health problems. The body may then attempt to counter this negative path by increasing testosterone release, which may explain why females with clinical health conditions such as obesity and breast cancer have higher testosterone levels than expected, given their age [4, 10, 14]. Given that the research in this area is in its infancy and many studies have methodological flaws (i.e., small sample sizes, even smaller samples of females), these assertions are highly speculative and theoretical.

Examination of each of the individual health behaviors through post hoc analyses revealed that positive affect and salivary testosterone only predicted engaging in a regular exercise routine, but not fruit/vegetable intake or hours of sleep at a *p* value less than .05. Though the sample size of this study (*n* = 87) is fairly large for this area of work [5], a larger sample would provide greater power to detect differences [38]. Of note, the *p* value of the interaction between testosterone and positive affect was .06 in the model predicting exercise, and the interaction between testosterone and positive affect was significant (*p* = .047), but the model was not (*p* = .12) in predicting fruit and vegetable intake. This area of study would be strengthened by increasing the sample sizes and diversity of participants across age, setting, gender, reproductive status, and ethnicity, thus increasing the representativeness of the results. Given that the study also identified differences in relationship status and oral contraceptive use, both of which have been documented in previous studies [5], it would be advantageous to have large enough samples to conduct separate analyses or interactions among these variables as well.

In addition to the hypotheses examined, it is also important to note that there was a significant difference in testosterone levels within the sample by socioeconomic status such that individuals who reported an income of less than $10,000 per year also had significantly lower levels of testosterone than those who reported an annual income of $10,000 or more. The present study includes a sample of racially diverse, lower income university students. If testosterone is conceptualized as the search for or maintenance of social status [8], these individuals are likely to experience stress as a result of their lower rank in social status. Though these individuals attend university and likely have their basic needs met, they perceive themselves to be poor or less advantaged. As Sapolsky [39] describes, poverty alone is not what predicts poor health outcomes, but the perception of poverty among others who have much more. There is not yet a clear understanding of psychosocial outcomes associated with low levels of testosterone in women or what health implications these findings may have, but it certainly needs to be explored further.

Though the model examined in this study implies a causal direction, it is important to note that the data is cross-sectional and was taken at one time point. It is possible that health behaviors influence testosterone (likely keep testosterone levels at a moderate range) and perhaps increase positive affect (though not to a level that might lead to maladaptive levels of emotions, warranting clinical attention). As no studies have examined the interplay among these three important health variables, it is difficult to draw meaningful conclusions. A large, randomized-controlled design in a sample of healthy female participants with four groups assigned to either alter testosterone, positive affect, health behaviors, or no change would be necessary to draw causal conclusions. As this data represents a preliminary investigation of the connections between testosterone, affect, and behavior, additional cross-sectional and experimental research is warranted.

The study has a number of limitations not yet addressed. Potential confounds, such as food intake, medication use, and substance use, were purely based on self-report of participants. Minimal exclusion criteria were implemented and the study took place across two academic years at two time points to increase sample size and accommodate student involvement at a liberal arts university. An oral swab was used to increase saliva production and samples were analyzed using immunoassays, both of which have been called into question [11]. Our sample reported a mean salivary testosterone level of 44.68 pg/mL (*SD* = 23.54, range 6.93–164.79), which is within the range of the manufacturer’s recommendations and consistent with population-based studies of salivary testosterone using liquid chromatography-tandem mass spectrometry [10]. It is important to note that hormone research requires estimation and exact measures may never be possible; however, the advantages of salivary testosterone assessment far outweigh the limitations, given its ease and access [5].

The health behavior composite used to assess health-promoting behaviors is both a strength and limitation of the study. Assessing behavior is always a challenge, particularly when relying on self-report to do so. One of the challenges in assessing health behavior is that there is no gold standard agreed upon method to assess health behaviors across multiple domains. Though single-item questions of health behavior have been shown to be powerful predictors of health outcomes (i.e., [40]), health behaviors appear to have a cumulative effect on mortality (i.e., [26]) and to focus on one is insufficient. The use of a composite measure is statistically advantageous as it allows for participants to “get credit” for engaging in any behavior, rather than examining each separately, thus decreases the number of tests run and type 1 error rate [29]. The composite score was also normally distributed across behaviors, while the number of participants who did or did not engage in various behaviors (i.e., cigarette use) would have made it impossible to include this important health behavior in the analyses. In addition, some of the items are not clearly defined (i.e., eat 3–5 servings of fruit/vegetables), and it is left up to the participant to decide what it means. For practical purposes, participants are more likely to know if they ate a banana versus whether they consumed 80 g of fruit. Again, it is an estimate and endorsing “yes” may just mean that they know it is important and they attempt to engage in this behavior. Regardless, additional reliability and validity studies of the health behavior assessment used or development of one is warranted and would be useful in future studies.

At first glance, the sample seems healthier than what would be expected of this age group. According to the most recent data of the Behavioral Risk Factor Surveillance System [41], an annual survey conducted by the Centers for Disease Control and Prevention, among adults ages 18–24 in the state where the current study was conducted, 93.4% report that they do not smoke, 27.7% report that they consume five or more servings of fruits/vegetables a day, and 84.3% report that they participated in physical activity over the last month. Though the current sample was fairly healthy (98% do not use cigarettes, 62% have a regular exercise routine, and 44% consume 3–5 servings of fruit/vegetables per day), they do appear to be representative of the geographical location from which they were sampled. It would be useful to sample across geographical locations in future studies.

Analysis of data in only females, with the exclusion of male participants, is also both a strength and limitation. The data was collected at a small, liberal arts institution where teaching is a priority. As such, all interested students were welcome to participate to enhance their learning experience, with the plan to screen for known outliers or covariates. Given the well-documented differences in male and female participants in salivary testosterone research (i.e., [5]), separate analyses for males and females are necessary. Only 18 males participated, which does not provide enough power to test the relationships of interest in men. Historically, biomedical research has not included female species due to variability that is often not supported by research (i.e., [42]). This has led to a deficiency in our understanding of female biology. Until recently, women were avoided in studies of testosterone in order to avoid confounds such as menstrual cycle or a belief that testosterone does not play an important role in women’s health, despite evidence that controlling for menstrual cycle is unnecessary unless this is a target variable of the study (i.e., [5]). When females have been included in testosterone research, it is primarily in sexuality and clinical studies where hormone levels are expected to be significantly different (i.e., [5]). The primary reason that validity of salivary testosterone in women has been described as a methodological issue may be because it is always compared with assessment in men and, therefore, may be held at an unrealistically high standard. Comparing gender/sex differences may not be an accurate or appropriate way to analyze data that includes testosterone, particularly salivary testosterone, given what we do know about the differences detected between men and women.

Though a preliminary investigation, the current study provides insight into the interplay among neuroendocrine function, psychological factors, and behavior in a relatively large sample of female university students and includes racial and socioeconomic diversity uncharacteristic of much of the data collected using these variables. Given the importance of testosterone in psychological and physical health outcomes for both men and women, it is essential to increase investigation of the role that testosterone plays in women’s health.
